# Stepwise implementation of a cardiovascular risk management care program in primary care

**DOI:** 10.1186/s12875-021-01602-w

**Published:** 2022-01-05

**Authors:** Geert Smits, on behalf of the PoZoB primary care group, A. Romeijnders, H. Rozema, C. Wijnands, Monika Hollander, Sander van Doorn, Michiel Bots

**Affiliations:** 1 Bolwerk 11-14 5509, MH Veldhoven, The Netherlands; 2 Julius Center for Health Sciences and Primary Care, University Medical Center Utrecht, Heidelberglaan, 100 3584 CX Utrecht, The Netherlands

**Keywords:** Primary care, General practice, Cardiovascular disease, Programmatic care, Practice nurse, Implementation

## Abstract

**Background:**

Primary care plays a pivotal role in sustainable cardiovascular risk management (CVRM) but little is known about the organizational process of implementing the guidelines. The aim of the study was to describe the approach taken by a primary care group to implement the CVRM guideline.

**Methods:**

Stepwise introduction and implementation of a programmatic CVRM care program was organized and facilitated by the care group between April 2010 and January 2013 in 137 affiliated general practices with 188 general practitioners (GPs), in the vicinity of Eindhoven, Netherlands. Care group support comprised sufficient staff, support with data extraction based on ICPC and ATC codes and with identification of eligible patients by scrutinizing patient health records and adequate coding of disease conditions.

**Results:**

Patient selection based on availability of structured information on ICPC codes and risk factor levels from the electronic health records, led to 38,675 eligible patients in 2013. December 2019, the CVRM program was still running in 151 practices with 51,416 patients receiving programmatic CVRM care. Linking problems between 8 different electronic health record systems and the multidisciplinary information system for integrated care delayed adequate data collection until the beginning of 2013.

**Conclusion:**

Commitment of affiliated GPs, a structured approach with adequate coding of diagnoses and risk factors, central data registration and additional funding for sufficient staff support are important conditions for the introduction and implementation of successful and sustainable programmatic CVRM care. This approach constitutes the basis for long-term follow up and annual evaluation.

**Supplementary Information:**

The online version contains supplementary material available at 10.1186/s12875-021-01602-w.

## Background

Cardiovascular diseases (CVD) are the most important cause of morbidity and mortality in low, middle and high income countries [1, 2]. The most important modifiable risk factors for CVD are smoking, unhealthy lifestyle (alcohol consumption, unhealthy diet, physical inactivity), overweight, elevated blood pressure, unfavorable lipid levels and diabetes mellitus. These are considered to be responsible for 80–90% of preventable CVD [3]. National and international guidelines have been published to recommend approaches for prevention [4–6]. According to the Prevention Guideline of the European Society of Cardiology the general practitioner (GP) has a unique role in identifying individuals at risk for CVD and assessing their eligibility for intervention based on their risk profile” [7].

In the Netherlands many GPs are joint together in local or regional ‘care groups’. A care group is an organization with a legal entity in which health care providers work together. The care group is responsible for the coordination and provision of contracted care in a particular region [8]. Care groups negotiate with regional health insurers about a bundled payment contract/integral funding. By joining a care group, practices benefit from a negotiated renumeration, support with implementation and regular education for health care providers. Care groups also organize chronic care programs for several conditions, such as diabetes mellitus type 2 (DM2), asthma/COPD (A/C) and cardiovascular risk management (CVRM). These chronic care programs are performed by a registered nurse with an additional training, in close collaboration with a GP. The Primary Care group PoZoB (Praktijk ondersteuning Zuid-oost Brabant) originated from an initiative of 6 smaller GP collectives, to which many other GPs voluntarily joined. PoZoB started with the implementation of chronic care programs for DM2 and A/C in 2005 and 2008 respectively, followed by CVRM in 2010. The content of the CVRM care program was based on the 2006 CVRM guidelines of the Dutch Society of General Practice [4]. The guideline provided the GP with recommendations on who to screen but made no recommendations on how to organize screening. Furthermore, the effect of strict application of the guideline in terms of cardiovascular risk control and prevention of vascular events in general practice had not been evaluated. This provided the ‘research gap’ at the start of the study. To the best of our knowledge, we are among the first describing the introduction and stepwise implementation of a carefully designed cardiovascular risk management program offered by a primary care group.

## Methods

### Study population

At the start of the CVRM implementation project in 2010, the care group comprised 137 GP practices (188 GPs) with 402,623 registered patients living in the south-eastern part of the province of Brabant, and the western part of the province of Limburg in The Netherlands.

### Conditions for starting

Before starting the implementation process, the PoZoB primary care group contacted every individual practice to identify suitability for participation in the CVRM care program, defined as i) participation in running chronic care programs for DM2 and A/C, and ii) having sufficient hours deployed for the practice nurse (PN) and the GP. The number of hours needed was estimated by the care group based on the number of participating patients, the number of annual consultations and the consultation time. If these conditions were met, the implementation process could start. Implementation start was linked to quarterly renumeration, therefore practices started every 3 months. The implementation start of 137 practices (188 GPs) quarterly is given in Table 1.

**Table 1:** Practices and GPs starting implementation programmatic CVRM care

Year	2010			2011				2012			
Quarter	Q2	Q3	Q4	Q1	Q2	Q3	Q4	Q1	Q2	Q3	Q4
Practices (n)	26	0	16	15	26	24	20	4	2	2	2
GPs (n)	41	0	19	16	35	29	32	7	3	4	2

### Study design

We designed the study as a non-experimental prospective cohort study embedded in routine clinical practice.

### Implementation steps that were taken

The stepwise implementation comprised 4 steps: 1) Data extraction; 2) Inclusion/exclusion of eligible patients; 3) Consultation; 4) Follow up.

1. Data extraction.

Data extraction occurred from the patient electronic health record (EHR) to identify an increased risk for cardiovascular disease or with a history of cardiovascular disease. Extraction of data was carried out by a certified organization for multidisciplinary data management and scientific research (“Meetpunt Kwaliteit”, since 2015 INZO = Instituut voor ZorgOptimalisatie). Potential eligibility for the program was based on medical diagnosis, and/or prescribed medication in patients older than 18 years and/or on labels that practices already used for identifying patients. Medical diagnosis was linked to the International Classification of Primary Care (ICPC) code and prescribed medication was linked to the Anatomical Therapeutical Chemical (ATC) classification. The ICPC and ATC codes used for identification are listed in Supplementary Table 1.

2) Inclusion/exclusion of eligible patients.

The PN plays an essential role in step 2, 3 and 4 of the implementation process. For in- and exclusion, the EHR was scrutinized by the PN if diagnoses or risk factors were set correct, based on discharge letters from the specialist. Practices were given 1 year to complete the EHR investigation for correct ICPC codes and labeling, invite patients and start with CVRM care. A mandatory group course was offered to the GP and PN to standardize registration of patients. During this year, all practices were visited two or three times by specialized care group nurses to monitor progress and to discuss unclear diagnoses. If necessary, staff nurses were reachable by phone to discuss in- or exclusion in the CVRM care program.

Criteria for inclusion and exclusion in the CVRM care program were: (1) Patients with CVD or kidney disease: (i) documented ischemic or atherosclerotic heart disease (myocardial infarction and angina pectoris), heart failure, atrial fibrillation, aneurysm of the abdominal aorta, peripheral arterial disease, transient ischemic attack, ischemic or hemorrhagic stroke, chronic kidney disease and (ii) primarily treated in primary care and (iii) aged 18 years or above. (2) Patients without a history of CVD or kidney disease but at high risk of CVD (i) a 10 year cardiovascular mortality risk > 5%, based on the SCORE table from the 2006 CVRM guidelines of the Dutch Society of General Practice (4) or (ii) use of blood pressure lowering or lipid lowering drugs in men aged ≥55 years and women aged ≥60 years or (iii) Systolic blood pressure > 180 mmHg and/or total cholesterol > 8 mmol/l ever measured, independent of the 10 year mortality risk. Furthermore, these patients needed to be primarily treated in primary care and aged 18 years or above.

Exclusion criteria for both groups were: (i) primarily treated by a specialist in a hospital or at an outpatient clinic or (ii) diabetes mellitus (as they received cardiovascular risk management in a diabetes care program). Criteria are based on the CVRM guidelines of the Dutch Society of General Practice [4].

Those eligible were flagged in the EHR using the *Care2U system.* In 2010 a Multidisciplinary Information System for integrated care (Care2U) was introduced. Care2U data registered by the PN ended up automatically in the EHR. All patients were labeled in Care2U and in the EHR as follows:

- V1: Patients at high risk for CVD, treatment and follow up by the GP/PN
- V2: Patients at high risk for CVD, treatment and follow up by a specialist in hospital or out-patient clinic
- V3: Patients at high risk for CVD, refusing care by a GP/PN
- Z1: Patients with CVD, treatment and follow up by the GP/PN
- Z2: Patients with CVD, treatment and follow up by a specialist in hospital or out-patient clinic
- Z3: Patients with CVD, refusing care by a specialist

Ultimately, only patients who had their treatment and follow up in primary care (V1 and Z1) were included in the CVRM care program.

3) Consultation

Once a patient was eligible for CVRM care he/she received an invitation letter from the general practice to make an appointment with the PN. If the patient did not respond within 2 weeks, the practice assistant called the patient. During the first consultation the patient was asked whether he/she was willing to participate in the CVRM care program. Time for the intake was estimated 45 min and was often split in 2 visits to the practice. The intake comprised an interview, check of in- and exclusion criteria, a physical examination and referral for blood testing to the local laboratory to complete the cardiovascular risk profile. The main items from the interview the PN conducted with the patient are summarized in Table 2.

**Table 2:** Summary of the aspects addressed during PNs interview

• Physical, psychological and social well-being, stress related problems, sexual problems	
• Cardiovascular (familial) history: 1st degree family member (< 60 years) with previous CVD	
• Cardiovascular complaints:	
- Chest pain	
- Shortness of breath	
- Palpitations	
- Headache	
- Blurred vision	
- Fatigueless	
- Swollen ankles	
- Leg pain while walking	
• Daily food intake:	
- Salt intake less than 6 g a day	
- 200 g of vegetables a day and 2 pieces of fruit	
- Eat fatty fish twice a week	
- Reduce saturated fat intake	
- Alcohol intake	
• Smoking	
• Exercise 30 min a day, 5 days a week	
• Laboratory results	
• Cardiovascular medication use	
- Adherence (based on patients estimate how often medication was forgotten every week)	
- Possible side effects and interactions	
- New prescription(s)	

The protocolized physical examination, based on the CVRM guidelines of the Dutch Society of General Practice, assessed blood pressure, height and weight and body mass index (BMI kg/m^2^), heart rate and waist circumference. Blood and urine samples were taken for fasting glucose, lipids (total cholesterol, HDL, LDL and triglycerides) and kidney function (serum creatinine, estimated glomerular filtration ratio (eGFR) and proteinuria). Blood tests performed less than 3 months ago could also be used. Blood sampling was performed at a local hospital laboratory or at a local diagnostic health center. Based on test results, for patients without a history of a cardiovascular disease and without preventive cardiovascular medication, a 10 year cardiovascular risk was estimated using the Systematic Coronary Risk Evaluation (SCORE) table from the 2006 CVRM guidelines of the Dutch Society of General Practice (4). For patients younger than 55 (men) or 60 (women) years of age the SCORE had to be calculated based on blood pressure or cholesterol levels before starting medication. If the SCORE was ≤5%, the patient was not eligible for the CVRM care program.

4) Treatment and follow-up

After determining the cardiovascular risk profile, the PN made an individual care plan with the patient and supported self-management by informing about CVD or risk factors and motivating the patient to take the lead in coping with it in the best possible way. The PN discussed the individual care plan with the GP, who had final medical responsibility. The PN was responsible for preparing and discussing the individual care plan with the patient, comprising non-pharmacological and pharmacological treatment. Although treatment goals were set in shared decision with the patient, in most cases targets values were used according to the 2006 CVRM guidelines of the Dutch Society of General Practice (4), which are listed in Supplementary Table 2.

If necessary, the PN referred the patient to a smoking cessation program, a physiotherapist or a dietician. If lifestyle advise resulted in insufficient effects on the risk factors level, blood pressure or lipid lowering medication could be initiated after consultation with the GP. When a cardiovascular event occurred or a patient failed to meet target values for blood pressure or lipids after maximum drug therapy, the GP referred to or consulted a specialist according to the criteria outlined in Table 3.

**Table 3:** Referral recommendations

Criteria for consultation of a nephrologist:	
• Patients < 65 years with a eGFR 45–60 ml/min/1,73 m2	
• Rapid deterioration of the renal function (> 3 ml/min/year)	
• Patients > 65 years with a eGFR 30–45 ml/min/1,73 m2	
• Increase of albuminuria despite adequate pharmacological treatment	
Criteria for referral to a specialist	
• A new cardiovascular event	
• Failure to meet target values despite adequate medication	
• Familial dyslipidemia	
• Premature, familial or undefined vascular disease	
• Suspicion of secondary hypertension.	
• Hypertension emerged in a short time and at young age (< 35 years)	
• Suspected malignant hypertension (diastolic blood pressure > 120 mmHg or clinical manifestations appropriate to cerebral complications like reduced consciousness, delirium, confusion, sudden impairment of vision or epileptic phenomena)	
• Macro-albuminuria (Albumin-Creatinine Ratio > 30 mg/mmol) and/or eGFR (< 30 ml/min/1,73 m2)	
• Patients with suspected underlying kidney disease, familial kidney disease or specific sediment abnormalities	

Regular follow up was agreed with the patient, depending on presence of comorbidity, complexity, achievement of treatment goals and advice according to the CVRM guideline. Patients were seen at least once a year by the GP to discuss laboratory results and seen 1–3 times a year by the PN to discuss lifestyle improvement and setting of new treatment goals if needed or wanted. In order to involve practices as much as possible in the development of the CVRM care program, the care group organized annual feedback meetings to discuss results and regular education for GPs and PNs on cardiovascular-related subjects.

## Results

### Data collection and registration

In 2010 patient data (anamnestic, biometric, laboratory) were collected in a multidisciplinary registration system for integrated care (Care2U). Data registered in Care2U automatically ended up in the patients EHR and was accessible to the GP. Care2U automatically called patients for annual blood tests by linking the call to the date of birth. Due to registration problems in the EHR and to linking problems between 8 different EHR systems and Care2U, data collection was delayed and limited at the beginning. In the first quarter of 2013 these problems were finally solved. From April 2013, Care2U data were monitored monthly and every practice was able to view their data (process and outcome) any time.

### Patients participating in programmatic CVRM care

On April 1st 2010, 30 practices (41 GPs) with 87,458 registered patients started with the selection process which led to the identification of 11,891 patients suitable for integrated CVRM care (13,6% of the registered patients). Because 400 patients (0,5%) eligible for primary prevention, decided not to take part in the program and for 3037 patients (3,5%) cardiovascular care was delivered in hospital or outpatient clinic, 8454 patients (9,7%) participated in the CVRM care program: 5988 high risk patients (6,8%) and 2466 CVD patients (2,8%). Due to quarterly renumeration, practices could start implementation every 3 months. In January 2013, 137 practices and 38,675 patients had started with programmatic CVRM care. Another 8 practices started with programmatic CVRM care in 2013, 2014 and 2015. Between 2016 and 2019 6 new practices affiliated to the care group. The number of practices and GPs, eligible patients and baseline characteristics of participating patients between 2010 and 2019 are listed in Table 4.

**Table 4:** Number of practices/GPs, eligibility and baseline characteristics of participating patients

Year	2010	2011	2012	2013	2014	2015	2016	2017	2018	2019
Practices (n)^a^	42	122	137	140	143	145	148	149	151	151
GPs (n)^a^	60	172	188	191	194	196	199	200	202	202
Registered pat^a^	87,458	378,099	402,623	406,119	416,433	422,296	401,077	405,956	422,452	436,009
Eligible pat^a^	11,896	50,937	54,990	61,709	63,846	65,692	64,877	65,166	67,194	68,989
(%)	13,6	13,5	13,7	15,2	15,3	15,6	16,2	16,0	15,9	15,8
Particip pat^b^	8454	34,630	38,675	39,504	42,554	45,139	48,222	46,400	48,397	51,416
(%)	9,7	9,2	9,6	9,7	10,2	10,7	12,0	11,4	11,5	11,8
Age (mean)^b^	±	67,2	67,8	68,1	68,5	68,9	69,5	69,5	69,8	70,1
Male (%)^b^	±	47,1	47,4	46,9	47,2	47,3	47,9	48,2	48,7	48.9
Second prev (%)^b^	29,2	28,6	33,3	35,3	37,0	38,2	40,6	43,9	44,3	45,2
≥ 70 Years (%)^b^	±	42,6	43,6	44,7	46,2	47,6	50,5	51,5	52,8	54,3

^a^Numbers from accountability reports (2010–2015) and quarterly reports (2016–2019)

^b^Numbers from Care2U data (2010–2019)

± Incomplete data

Second prev: Patients eligible for CVRM care with previous CVD

### Facilitating factors and barriers for implementation

Facilitating factors for implementation were i) sufficient time given to PNs to provide patients with correct ICPC coding, ii) mandatory education for PNs and GPs on scrutinizing the EHR and iii) practice visitations by care groups’ staff members every 3 months to discuss progress. Because all affiliated practices had given commitment, there were basically no serious barriers for implementation. Practicability in daily practice is mainly determined by sufficient time and care group support.

## Discussion

### Summary

This paper describes the implementation of a CVRM care program in 137 practices within less than 3 years for 38,675 patients, a number that increased to 51,416 patients in 2019, comprising around 11% of patient GP population. Facilitation factors were interest and commitment of GPs, sufficient allocated time for tasks, education linked to regular feedback and monitoring and a program based on approved guidelines.

### Comparison with existing literature

The gap between evidence and real world practice has been identified long ago [9]. Yet, the evidence based on the best design and the best implementation strategies for prevention of cardiovascular disease is still limited. Already in 2010, Grol and co-workers, showed that commitment for chronic care, a stepwise and structured approach, sufficient staff support and implementation with regular feedback, are important facilitators [10]. This has recently been confirmed again by Wandell and co-workers that lack of time and renumeration were amongst the most important barriers for selective prevention of cardiovascular diseases [11]. Organizational changes in patient care, in terms of enhancing performance of non-physicians (practice nurses), computer systems integrated in the electronic health care records networks of the GPs, allow for automated reminders and clinical decision support have been known to be effective in improving patient care with reduced costs and improve patient outcomes with multidisciplinary teams and integrated care services [12]. Standard registration, feedback loops with training followed by improvement strategies and education as part of a learning health care system has been advocated. Our approach developed and applied in 2010 by deploying PNs and negotiating an adequate reimbursement, addressed most of these issue and perhaps therefore, experienced little barriers. In addition the our approach, a recent review from the SPIN-EU group came up with an evidence based generic “toolbox” for circumventing identified obstacles and harnessing facilitators in the design and implementation of cardiovascular prevention strategies. The development of the toolbox was based on data from five European countries, and may be of use in different communities, countries, and cultures [13].

### Strengths and limitations

With all participating practices being affiliated to the care group, optimal reach, adoption and implementation of the CVRM program was assured. All practices in the Zuid-Oost Brabant region in the Netherlands were affiliated with the PoZoB care group. Practices covered rural, suburban and urban areas similar to the rest of the Netherlands, and can therefore be considered a representative sample of Dutch GPs.

The 2006 CVRM guidelines of the Dutch Society of General Practice has provided criteria to identify high risk patients [4]. When using structured files in the EHR as primary source for identification of eligible patients, the completeness and correctness relies on the ICPC and ATC coding of patients performed in general practice. The proportion of potentially eligible patients per practice based on ICPC and ATC codes varied between 14.8 and 30.3%, suggesting that inadequate coding may exist, potentially leading to a considerable number of high risk patients who unfortunately remain out of sight. This is in line with an evaluation in 2012 of ICPC coding in 311 Dutch practices showing that ICPC coding varied substantially between practices and between EHRs [14]. We emphasized that adequate coding of disease and medication is an important prerequisite for implementation of a CVRM program in general practice. Another consideration is that most patients (men ≥55 y, women ≥60 y) with prescribed medication for hypertension and hypercholesterolemia were automatically included in the CVRM care program, while we did not know whether de diagnosis hypertension and hypercholesterolemia was set correctly according to the National CVRM Guideline [4], which may have led to overrepresentation. Monitoring the CVRM care program by means of quarterly reports ensures that results can be followed closely and adjusted if necessary. This is in line with the principles of the “Learning Healthcare System” (LHS) in which daily care data from electronic health records are compared and discussed to create continuous learning and improved health care delivery [15].

### Implications for research and/or practice

Using a Multidisciplinary Information System for data collection gives the opportunity for monitoring (i) the development of the CVRM care program and (ii) performance of individual practices based on process and outcome indicators over a long period of time. With annual collection of biometric and laboratory data it is possible to assess improvements in cardiovascular risk factors and reduction in cardiovascular events. In feedback meetings GPs and PNs were able to discuss further development of the care program and in meetings between PNs and nurse staff experiences were exchanged. As such, evaluation follows a qualitative approach according to the RE-AIM framework, which is based on 5 elements (Reach, Effectivity, Adoption, Implementation, Maintenance) for assessing the impact of innovation on individual and organizational level [16, 17]. The CVRM care program provides a solid basis for scientific evaluation on registration and outcomes of CVRM care and its determinants to explore practice variation and identify modifiable factors for improvement. Real-world data, which are becoming increasingly important in providing evidence of treatment effectiveness in clinical practice, allow us to evaluate the effect of our program in terms of improved cardiovascular outcomes and reduced cardiovascular events, and against which costs.

## Conclusion

In conclusion, commitment of affiliated GPs, a structured approach with adequate coding of diagnoses and risk factors, central data registration and additional funding for sufficient staff support are important conditions for the introduction and implementation of successful and sustainable programmatic CVRM care. This approach constitutes the basis for long-term follow up and annual evaluation.

## Supplementary Information

**Additional file 1: Supplementary File 1.** ICPC and ATC codes used to identify individuals potentially eligible for entering the CVRM care program.

**Additional file 2: Supplementary File 2.** Guidelines for treatment of patients enrolled in the CVRM care program based on 2006 CVRM guidelines of the Dutch Society of General Practice.

## Data Availability

No patient data have been used for this paper.

## Abbreviations

PoZoB: Praktijkondersteuning Zuidoost Brabant
CVRM: Cardiovascular risk management
GP: General Practitioner
ICPC: International Classification of primary Care
ATC-codes: Anatomical Therapeutic Chemical-codes
CVD: Cardiovascular diseases
DM: Diabetes mellitus
COPD: Chronic Obstructive Pulmonary Disease
PN: Practice Nurse
EHR: Electronic Health Record
CIS: Chain Information System
SCORE: Systematic Coronary Risk Evaluation
LHS: Learning Healthcare System
REAIM: Reach Effectivity Adoption Implementation Maintenance
