# Supplementary material for: Stepwise implementation of a cardiovascular risk management care program in primary care
Source: BMC Prim Care. 2022 Jan 5;23:1. doi: 10.1186/s12875-021-01602-w (PMC8746647; doi:10.1186/s12875-021-01602-w)
Supplement: Supplementary file 1 — Additional file 1: Supplementary File 1. ICPC and ATC codes used to identify individuals potentially eligible for entering the CVRM care program. [file 12875_2021_1602_MOESM1_ESM.docx]

**Supplementary File 1:**  ICPC and ATC odes used to identify individuals potentially eligible for entering the CVRM care program.

*Risk factors for cardiovascular disease (ICPC):*

K85 high blood pressure

K86 high blood pressure without organ damage

K87 high blood pressure with organ damage

T82 obesity

T93 fat metabolic disorder

T 93.1 high cholesterol

T 93.2 other/combined fat metabolic disorder

P17 smoking

B 85.1 of B 85.4 impaired glucose tolerance

T90 diabetes mellitus

T 90.1 diabetes mellitus, type 1

T 90.2 diabetes mellitus type 2

T90.3 diabetes mellitus, non-specified

U99.01 kidney disease

U98.01 proteinuria

A 29.1; A29.2; A29.3; A29.4 family history of cardiovascular disease

*Prevalent Cardiovascular diseases (ICPC):*

K74 angina pectoris

K75 acute myocardial infarction

K76 chronic ischemic heart disease

K91 atherosclerosis

K77 heart failure

K78 atrial fibrillation

K79.2 ventricular tachycardia

K83 non rheumatic valve disease

K84.02 cardiomyopathy

K89.1 transient ischemic attack

K90. stroke

K90.1 ischemic stroke

K90.2 hemorrhagic stroke

K92.1 peripheral arterial disease

K99.1 aneurysms aortae

ATC codes

*Medication (ATC):*

A10 anti diabetic medication

B01A Antithrombotic

B01AA Vit K antagonists

B01AC platelet aggregation inhibitors

C01 cardiac

C01AA digitalis glycosides

C01B anti-arrhythmic medication

C01DA nitrates

C02 antihypertensive medication

C03 diuretics

C04 vasodilatory medication

C07 beta-blockers

C08 calcium channel inhibitors

C09 RAAS inhibitors

C10 cholesterol lowering medication
