# Supplementary material for: Stepwise implementation of a cardiovascular risk management care program in primary care
Source: BMC Prim Care. 2022 Jan 5;23:1. doi: 10.1186/s12875-021-01602-w (PMC8746647; doi:10.1186/s12875-021-01602-w)
Supplement: Supplementary file 2 — Additional file 2: Supplementary File 2. Guidelines for treatment of patients enrolled in the CVRM care program based on 2006 CVRM guidelines of the Dutch Society of General Practice. [file 12875_2021_1602_MOESM2_ESM.docx]

**Supplementary File 2:** Guidelines for treatment of patients enrolled in the CVRM care program based on 2006 CVRM guidelines of the Dutch Society of General Practice .

*Non pharmacological treatment:*

- Referral to a smoking cessation program if indicated
- In case of overweight: aim for a BMI < 25 kg/m^2^
- Exercise: aim at moderate exercise at least 30 minutes a day, 5 days a week
- Maximum alcohol intake: 2 units a day for women, 3 units a day for men
- Nutrition:

- less than 10 energy percent saturated fat and less than 1 energy percent transfat

- preferably twice a week fatty fish

- at least 200 grams of vegetables a day and 2 pieces of fruit

- not more than 6 grams salt a day

*Pharmacological treatment with blood pressure and/or cholesterol lowering medication:*

- Patients without (a history of) cardiovascular disease
- Patients with a 10 year mortality risk > 10 %: consider pharmacological treatment
- Patients with a 10 year mortality risk of 5-10%: consider pharmacological treatment if one or more additional risk factors are present like familial burden of cardiovascular disease
- Patients with a systolic blood pressure > 180 mmHg or a Total Cholesterol > 8 mmol/L are always treated regardless of the Risk SCORE
- For smokers, stopping smoking is preferable to starting medication
- Patients with a (history of) CVD and a systolic blood pressure > 140 mmHg are treated with antihypertensive medication
- Patients with a history of stroke or TIA: consider anti-hypertensive treatment even with systolic blood pressure < 140 mm Hg
- In patients with angina pectoris, heart failure or experienced myocardial infarction a beta blocker is indicated
- In all patients with experienced CVD acetylsalicylic acid is prescribed, unless there is indication for oral anticoagulant therapy (atrial fibrillation, valve replacement)
- In all patients with experienced CVD cholesterol lowering therapy is advised

*Treatment targets according to the 2006 CVRM guidelines of the Dutch Society of General Practice*

Without CVD With CVD

Systolic BP < 140 mm Hg < 140 mm Hg

Diastolic BP < 90 mm Hg < 90 mm Hg

Total Cholesterol < 6,5 mmol/L < 4,5 mmol/L

LDL Cholesterol reduction ≥ 1 mmol/L < 2,5 mmol/L

BMI < 25 kg/m2 < 25 kg/m2

Waist circumference Women: < 80 cm < 80 cm

Men: < 94 cm < 94 cm

Fasting glucose < 6 mmol/L < 6 mmol/L

Potassium 3,5-5,0 mmol/L 3,5-5,0 mmol/L

Creatinine Women: 60-100 umol/L 60-100 umol/L

Men: 75-110 umol/L 75-110 umol/L

Creatinine Clearance (eGFR) > 60 ml/min/1,73m2 > 60 ml/min/1,73m2
